# Immunohistochemical demonstration of alteration of β-catenin during tumor metastasis by different mechanisms according to histology in lung cancer

**DOI:** 10.3892/etm.2014.2095

**Published:** 2014-11-28

**Authors:** XIANHUA XU, JI EUN KIM, PING-LI SUN, SEOL BONG YOO, HYOJIN KIM, YAN JIN, JIN-HAENG CHUNG

**Affiliations:** 1 Department of Pathology, Jilin Cancer Hospital, Changchun, Jilin 130012, P.R. China; 2 Department of Pathology, Seoul National University Boramae Hospital, Seoul 156-707, Republic of Korea; 3 Department of Pathology, Seoul National University Bundang Hospital, Seoul National University College of Medicine, Seongnam 463-707, Republic of Korea; 4 Department of Pathology, Presbyterian Medical Center, Jeonju 560-750, Republic of Korea

**Keywords:** β-catenin, wnt1, E-cadherin, lung cancer

## Abstract

The protein β-catenin exhibits a dual function in cells, by acting as a major structural component of cell-cell adherens junctions and as a central signaling molecule in the Wnt signaling pathway. However, how the regulation of β-catenin expression during tumor metastasis in non-small cell lung cancer (NSCLC) varies according to histological type remains unclear. To investigate the regulatory mechanism of β-catenin on tumor metastasis, the present study compared the expression of Wnt1, β-catenin and E-cadherin in 41 primary NSCLC tumors and their corresponding metastatic lesions by immunohistochemistry. Altered expression of β-catenin was more frequent in the metastatic tumors (34/41, 82.9%) than in the corresponding primary tumors (24/41, 58.5%; P<0.05). There were 12 cases [nine of adenocarcinoma (ADC) and three of squamous cell carcinoma (SqCC)] that revealed discordant β-catenin expression between the primary tumors and the corresponding metastatic lesions. Of these, 11 cases (11/12, 91.7%; nine ADCs and two SqCCs) demonstrated acquired β-catenin alterations in the metastatic lesions. Subgroup analysis of these nine ADCs revealed that six cases (6/9, 66.7%) were accompanied by E-cadherin loss but no Wnt1 overexpression. Subgroup analysis of the three SqCCs revealed discordant β-catenin expression. Two cases (2/3, 66.7%) demonstrated acquired β-catenin expression during metastatic progression with Wnt1 overexpression but no change in E-cadherin expression. One case of SqCC revealed normal β-catenin expression in the metastasis although the expression was aberrant in the primary tumor. The results of the present study revealed that the changes in β-catenin expression occurred during tumor metastasis by different mechanisms, depending on histological type. The alterations in β-catenin expression may be regulated by a cadherin-catenin system in ADCs with reduced membranous expression of E-cadherin, but mediated by Wnt1 overexpression in SqCCs with cytoplasmic or nuclear transition types.

## Introduction

Lung cancer remains the leading cause of cancer-associated mortality in developed countries, although certain clinically-relevant advances have been achieved (1,2). The majority of patients with lung cancer present with an advanced progression of the disease at the time of diagnosis and mortality is usually attributed to distant multiorgan metastases (3). Metastasis is a multistep process and ~90% of patients with cancer of any organ succumb due to metastases (4). Consequently, developing novel therapeutic strategies for the treatment of these patients presents a great challenge. However, there may be marked differences between the primary lung tumors and their metastases with respect to morphology and biomarker expression. A critical issue in the treatment of metastatic non-small cell lung cancer (NSCLC) is the genetic variability and differences between the primary tumors and their corresponding metastases.

The release of tumor cells from the primary tumor is the primary step of metastasis. Changes to cell adhesion molecules (CAMs) promote metastasis, which results in a deterioration of the disease prognosis. β-catenin, a multifunctional protein encoded in chromosome 3p21, is a crucial component of CAMs and is critical in cell-cell adhesion and tissue remodeling processes (5). In cell-cell adhesion, β-catenin binds to the intracellular domain of E-cadherin (6,7). E-cadherin is a well-characterized cell-cell adhesion molecule that connects neighboring epithelial cells in a specialized structure termed the adherens junction (8). It is an invasion suppressor gene that is frequently inhibited or undergoes mutation in invasive tumors (9). E-cadherin also mediates adhesive interactions between epithelial cells and exerts an effect on the organization of the actin cytoskeleton through binding to β-catenin.

β-catenin plays an important role in the Wnt/β-catenin signaling pathway by activating the transcription of target genes and leading to cell proliferation, invasion and metastasis (10). Wnt signaling is transduced through β-catenin, which is regulated by the adenomatous polyposis coli (APC)/axin/glycogen synthase kinase (GSK) 3β complex. In the presence of Wnt stimulation, the dishevelled segment polarity protein 1 (DVL1) gene is activated, at least in part by phosphorylation, which in turn recruits the GSK-3β complex away from the degradation complex (11–13). This allows the stabilization of β-catenin, resulting in the accumulation of free cytosolic β-catenin. β-catenin is able to translocate to the nucleus where it complexes with T cell factor (TCF)/lymphoid enhancer binding factor (LEF) to stimulate the expression of Wnt-target molecules (11,14).

Thus, the protein β-catenin exhibits a combined function in cells as a major structural component of cell-cell adherens junctions and as a central signaling molecule in the Wnt signaling pathway. However, few studies have analyzed the functions of β-catenin in patient-matched lung primary lesions and patient-matched distant metastases regarding either the cadherin-catenin system or the Wnt signaling pathway. In order to evaluate the dual role of β-catenin and to determine its importance as a predictor of metastasis and disease progression, the present study investigated the expression levels of Wnt1, β-catenin and E-cadherin in lung adenocarcinoma (ADC) and squamous cell carcinomas (SqCC) sections.

## Materials and methods

### Clinical data acquisition

The present study cohort comprised 460 patients with NSCLC who underwent surgical resection at Seoul National University Bundang Hospital (Seoul, Korea) from May 2003 to April 2008. Of the 460 patients included, 112 patients had recurrence and from these, 41 specimens of the primary tumors and their corresponding metastatic lesions were available for study. The current study procedures were approved by the Institutional Review Board (IRB) at Seoul National University Bundang Hospital (B1008-109-301).

### Immunohistochemical assays

Sections (4 μm thick) from formalin-fixed, paraffin-embedded tissue were deparaffinized in xylene and rehydrated in graded ethanol. The succeeding steps were performed automatically at 37°C using the Benchmark^®^ XT Slide Staining System Specifications (Ventana Medical Systems, Tucson, AZ, USA). Antigen retrieval was performed by immersing the slides in citrate buffer (pH 6.0) for 15 min and endogenous peroxidases were blocked with 1% H_2_O_2_ for 10 min.

The sections were incubated with rabbit polyclonal antibody for Wnt1 (H-89; Santa Cruz Biotechnology, Inc., Santa Cruz, CA, USA; dilution 1:100), mouse monoclonal antibody for β-catenin (5H10; Zymed Laboratories, Invitrogen Life Technologies, Carlsbad, CA, USA; dilution 1:1,000) and mouse monoclonal antibody for E-cadherin (SPM471; Thermo Fisher Scientific, Waltham, MA, USA; dilution 1:150) at 4°C overnight. The sections were subsequently washed with tris-buffered saline (pH 7.4).

The biotinylated secondary antibody was incubated with the sections for 20 min. The slides were then stained using a diaminobenzidine detection kit and counterstained with hematoxylin. The EnVision^™^ detection system (K5007, Dako, Glostrup, Denmark) was used which included a peroxidase-conjugated polymer backbone and secondary antibody molecules directed against rabbit and mouse immunoglobulins.

### Evaluation of the staining

Two pathologists (X.X and J.H.C.) independently performed blinded semiquantitative evaluations of the staining under a light microscope (BX51; Olympus Corporation, Tokyo, Japan), without prior knowledge of the patient data. To calculate the expression levels of Wnt1, the scoring criteria used were based on a semiquantitative approach, in which the percentage of positive tumor cells (0–100%) was determined and multiplied by the staining intensity (0, negative; 1, weak; 2, moderate; 3, strong). A total score within the range of 0–300 was generated for each sample, where 0–100 was classified as negative and 101–300 was classified as positive for Wnt1 expression.

For the expression of E-cadherin, a positive expression of <30% in the membrane was considered as reduced membranous expression. When evaluating the expression of β-catenin, a classification of staining patterns was used as follows: i) membranous pattern, the immunoreactivity was present solely in the cell membranes; ii) membranous-cytoplasmic pattern, the immunoreactivity was also present in the cytoplasm; iii) cytoplasmic pattern, the immunoreactivity was predominantly in the cytoplasm and in <20% of the nucleus; and iv) cytoplasmic-nuclear pattern, the immunoreactivity was present in the cytoplasm and concomitantly in >20% of the nucleus (15,16). Strong positively stained specimens of the cytoplasmic-nuclear, membranous-cytoplasmic and cytoplasmic patterns (≥10%) were considered positive (17). Specimens with membranous patterns <70% were considered to have reduced β-catenin expression (18).

### Statistical analysis

The analyses were performed using the SPSS 17.0 software (SPSS, Inc., Chicago, IL, USA). The χ^2^ test was used to evaluate the comparison of β-catenin, E-cadherin and Wnt1 expression between primary and metastatic tumors. P<0.05 was considered to indicate a medically statistically significant difference.

## Results

### Patient characteristics

Of the 460 patients, 112 patients (112/460, 24.3%) had recurrence and from these, 41 pairs of specimens of the primary tumors and the corresponding metastatic lesions were available for study. The origins of the 41 paired specimens used for analysis were the lungs (n=26), brain (n=6), bone (n=1), liver (n=1), pleura (n=3), spine (n=1), kidneys (n=2) and soft tissue (n=1; Table I). The patients consisted of 27 (65.9%) males and 14 (34.1%) females with ages ranging from 20 to 80 years (mean, 60.3 years). In terms of smoking status, the patients were divided into 19 (46.3%) non-smokers and 22 (53.7%) smokers. The hematoxylin and eosin-stained slides were reviewed independently by two pathologists (X.X. and J.H.C.) to confirm the original diagnoses, based on the World Health Organization criteria (19). There were 31 ADCs and 10 SqCCs, with the tumor diameters ranging from 1.5 to 8.0 cm (mean, 4.0 cm). The 41 patients were classified at the time of initial surgical removal using the 7th Edition of the International Union Against Cancer and American Joint Committee on Cancer (AJCC) TNM classification of Malignant Tumors from the International Association for the Study of Lung Cancer (IASLC) (20) as follows: pathological stage (P-stage) I, 9 (22.0%) patients; P-stage II, 14 (34.1%) patients and; P-stage III, 18 (43.9%) patients. The detailed clinicopathological characteristics of the patients in the present study are summarized in Table II.

### Expression of β-catenin by the primary tumors and the corresponding metastases

β-catenin expression was normally localized to the cell membrane of the respiratory epithelium; however, reduced expression in the cell membrane, cytoplasm and/or nuclear translocation was considered an indication of its aberrant expression. Altered expression of β-catenin was more frequent in the metastatic tumors (34/41, 82.9%) than in their corresponding primary tumors (24/41, 58.5%; P<0.05). In the 12 discordant cases of β-catenin expression, where the β-catenin expression status differed between the primary tumor and the metastasis, 11 cases revealed acquired β-catenin alterations in the metastatic lesions; the exception was one case of SqCC (case no. 39; Table III).

Following subgroup analysis of the 31 cases of ADC, discordance in β-catenin expression between the primary tumors and the metastatic lesions was revealed in nine cases (9/31, 29.0%). All nine discordant cases demonstrated acquired β-catenin alterations in the metastatic tumors (Table III).

**Figure 1:**
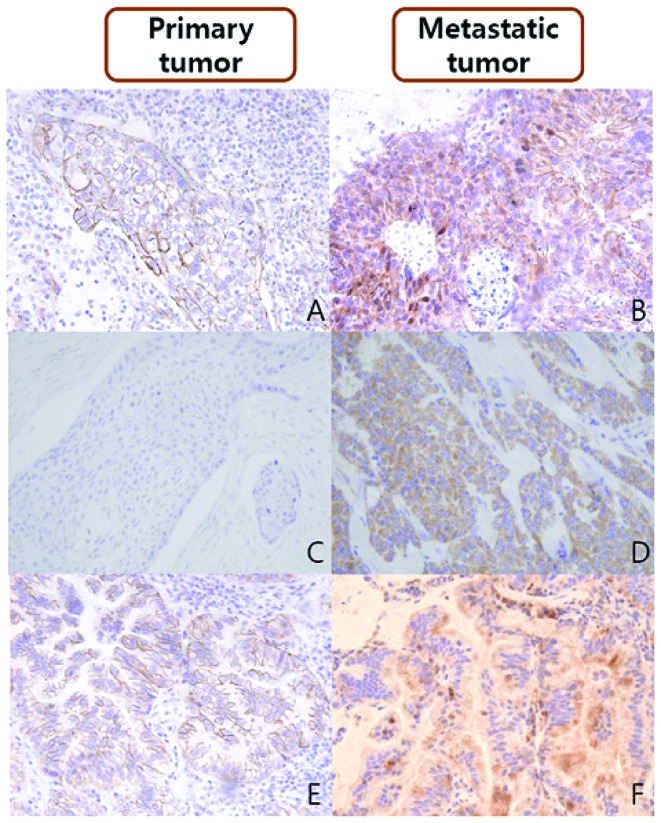
(A) The intact membrane expression of β-catenin in the primary tumor; (B) the alteration of β-catenin in the corresponding metastatic tumor; (C) no expression of Wnt1 in the primary tumor; (D) Wnt1 overexpression in the corresponding metastatic tumor; (E) the intact membrane expression of E-cadherin in the primary tumor and; (F) the membrane loss of E-cadherin in the corresponding metastatic tumor.

Subgroup analysis of the 10 cases of SqCC revealed three cases of discordance in β-catenin expression between the primary and matched metastases. Among them, two cases (case nos. 40 and 41) demonstrated acquired β-catenin alterations in the metastases but normal β-catenin expression in the primary tumors. The status of β-catenin expression in the primary and metastatic tumors is summarized in Fig. 1 and Tables III and IV.

### Correlation of β-catenin and E-cadherin expression in the primary tumors and the corresponding metastases

Immunohistochemical analysis revealed that E-cadherin expression was uniform in the cell membranes of the normal bronchial mucosa. E-cadherin expression was mainly observed on the membranes of the tumor cells and in certain cases, in the cytoplasm. Loss of E-cadherin expression had greater prevalence in metastatic tumors (26/41, 63.4%) than in their matching primary tumors (10/41, 24.4%; P<0.05; Fig. 1 and Table IV).

Following subgroup analysis of the 31 cases of ADC, discordance of E-cadherin expression between the primary tumors and metastatic lesions was observed in 17 cases (17/31, 54.8%). Among these, 15 (88.2%) cases acquired aberrant expression of E-cadherin in the metastases, although the expression was unaltered in the primary tumors. Altered β-catenin expression was associated with a decreased level of E-cadherin expression (P<0.001; Table V). Among the nine cases of ADC that demonstrated acquired β-catenin alterations in the metastatic lesions, six cases (6/9, 66.7%) were accompanied by E-cadherin loss but no Wnt1 overexpression (Table III).

In the subgroup analysis of the 10 cases of SqCC, there were five cases showing discordances in E-cadherin expression between the primary and matched metastases. Among these, four cases (case nos. 34–37) revealed decreased E-cadherin expression in the metastases but normal expression in the primary tumors (Table III). No significant association was identified between the expression of β-catenin and that of E-cadherin in the cases of SqCC.

### Correlation of β-catenin and Wnt1 expression in the primary tumors and the corresponding metastases

Wnt1 expression appeared in the form of a cytoplasmic staining pattern (Fig. 1). Wnt1 expression was negative in the non-neoplastic type I or II pneumocytes, bronchiolar epithelial cells, mesenchymal cells and inflammatory cells.

In the subgroup analysis of the 31 cases of ADC, discordance of Wnt1 expression between the primary tumors and metastatic lesions was observed in nine cases (9/31, 29.0%). Among the nine cases of ADC that revealed acquired β-catenin alterations in the metastatic lesion, only one case (1/9, 11.1%; case no. 25) had an accompanying change in Wnt1 expression. In a further seven cases (7/9, 77.8%) the expression levels of Wnt1 were preserved from the primary tumor to the metastatic lesions. The remaining case (1/9, 11.1%; case no. 20) overexpressed Wnt1 in the primary tumor but revealed no expression of Wnt1 in the metastatic tumor (Table III). No correlation was identified between the expression of Wnt1 and β-catenin in the cases of ADC (Table V).

In the subgroup analysis of the 10 cases of SqCCs, the expression of Wnt1 was preserved from the primary to metastatic tumors in 50% (5/10) of cases. There were three cases revealing discordance in β-catenin expression between the primary and matched metastases. Among them, two cases (case nos. 40 and 41) that demonstrated β-catenin alterations in the metastases and intact expression in their primary tumors exhibited acquired Wnt1 overexpression in the metastases. The remaining case (case no. 39) exhibited altered β-catenin expression in the primary tumors but intact β-catenin expression in the metastatic lesion. In these three cases, altered β-catenin expression was accompanied by altered Wnt1 expression, but no change in E-cadherin expression (Table III). However, no significant association was identified between the expression of β-catenin and Wnt1 in the primary and metastatic tumors due to the small number of cases examined.

## Discussion

The present study aimed to investigate whether the distant metastases of lung cancer may have aberrant β-catenin expression compared with the primary tumor and how such β-catenin expression is regulated. Distant metastases are common and numerous studies that have investigated metastatic patterns in primary lung cancer have revealed large variations in the frequency of metastatic involvement between organ systems (4,21,22). The most frequently involved intrathoracic sites are the: lungs (24–97%), mediastinal lymph nodes (46–85%) and pleura (15–45%). The most frequently involved extrathoracic sites are the: liver (38–58%), brain (14–45%), bone (20–40%) and adrenal glands (36–64%) (23,24). The data from the present study are within a similar range to those previously reported.

In the current study, aberrant expression of β-catenin in the primary tumors was preserved in the metastatic lesions for the majority of cases of ADC and SqCC (23/41, 56.1%), with the exception of one case of SqCC. The acquisition of changes in β-catenin expression during the metastatic progression of NSCLC were observed in 26.8% (11/41) of cases. These results suggest that the alteration of β-catenin expression is an important mechanism in tumor progression and the metastasis of NSCLC. Previous studies have also demonstrated that the aberrant expression of β-catenin is an independent prognostic factor of NSCLC, regardless of tumor, node and metastasis (TNM) stage (16,25).

The results of the present study also indicate that the loss of E-cadherin expression was much more prevalent in the metastatic tumors than in their matching primary tumors. Intact complexes of β-catenin/E-cadherin are important adhesion molecules and inhibitors of cancer invasion and metastasis. In the current study, the results indicate that changes in the stability and function of the β-catenin/E-cadherin complex resulted in the development of metastasis in tumors. A number of previous studies have also revealed a significant association between reduced E-cadherin expression and tumor differentiation grade, lymph node involvement, venous invasion and distant metastasis (24,26). However, few studies have verified these results using primary tumors and their matching metastatic tumors.

The results of the present study demonstrated that the alteration in β-catenin expression may be affected by signaling pathways that differ according to histological type. Alterations in β-catenin expression were significantly associated with the loss of E-cadherin in ADCs. Among the nine cases of ADC that acquired β-catenin alterations in the metastatic tumors, six cases (66.7%) were accompanied by a reduction in E-cadherin expression, and there was only one case (11.1%) in which Wnt1 overexpression was detected in the metastatic tumor. These results suggest that the aberration of β-catenin expression during tumor progression and metastasis in ADCs is more likely to be associated with the cadherin-catenin system than with the Wnt signaling pathway. Previous studies have also revealed that the aberration of β-catenin expression may induce epithelial-mesenchymal transition (EMT) during the development of cell lines and tumors (27–29). Another previous study has demonstrated that altered E-cadherin/β-catenin complex expression is important in tumor invasion and has an effect on the survival rate of patients with ADCs (30). Collectively, the results of present study revealed that the cadherin-catenin system is an important regulator of the distant metastasis of lung ADC.

Alterations in β-catenin expression between the primary and metastatic tumors in the cases of SqCC were accompanied by Wnt1 overexpression but not a loss of E-cadherin expression. This suggests that the aberration of β-catenin in tumor progression and metastasis in SqCCs may be associated with the Wnt signaling pathway rather than the cadherin-catenin system. However, it remains unclear as to whether the metastasis in SqCC is mediated by the Wnt signaling pathway due to the small number of cases analyzed. Therefore, further large-scaled studies are required to evaluate the role of the Wnt signaling pathway in the metastasis of SqCC.

The present study had certain limitations. The sample size was relatively small, particularly that which dealt with the Wnt-1 regulated β-catenin expression. Of the original 460 patients, 112 had recurrences but only 41 of these (31 ADCs and 10 SCCs) had tissue samples that were available for further study. Since the majority of patients with extrathoracic metastasis did not undergo further biopsies or surgery, the primary and corresponding metastatic specimens were scarce and difficult to obtain. Thus, larger studies are required to confirm the observations made in the current study.

In conclusion, three significant results were established in the present study. Firstly, primary lung carcinomas that develop into distant metastases may have aberrant β-catenin expression. Secondly, aberration of E-cadherin expression occurred frequently during the metastatic progression of NSCLC. Finally, changes in β-catenin expression may be regulated by the cadherin-catenin system in ADCs, but mediated by the Wnt pathway in SqCCs.

## Figures and Tables

**Table I:** Status of recurrence.

Metastatic site	Total (n)	Available tissues (n)
Lung	53	26
Brain	15	6
Bone	12	1
Liver	6	1
Pleura	12	3
Adrenal gland	3	0
Spine	3	1
Pancreas	2	0
Kidney	2	2
Soft tissue	2	1
Skin	1	0
Heart	1	0
Total	112	41

**Table II:** Clinicopathologic characteristics.

Characteristics	No. of patients	Percentage (%)
Total	41	100.0
Gender		
Male	27	65.9
Female	14	34.1
Age		
≤65	24	58.5
>65	17	41.5
Smoking		
Non-smoker	19	46.3
Smoker	22	53.7
Histological type		
Adenocarcinoma	31	75.6
Squamous cell carcinoma	10	24.4
Tumor size		
≤3 cm	13	31.7
>3 cm	28	68.3
Pleural invasion		
Absent	25	61.0
Present	16	39.0
Venous invasion		
Absent	31	75.6
Present	10	24.4
Lymphatic invasion		
Absent	15	36.6
Present	26	63.4
Perineural invasion		
Absent	37	90.2
Present	4	9.8
Necrosis		
<10%	22	53.7
≥10%	19	46.3
Tumor status		
T1	10	24.4
T2	24	58.5
T3	7	17.1
Nodal status		
N0	16	39.0
N1	8	19.5
N2	16	39.0
N3	1	2.4
P-stage		
stage I	9	22.0
stage II	14	34.1
stage III	18	43.9
Survival		
Alive	25	61.0
Succumbed	16	39.0

P-stage, pathological stage.

**Table III:** Immunohistochemical changes from primary to metastatic tumors.

No.	Histology	Metastasic site	β-catenin	E-cadherin	Wnt1
1	ADC	Lung	●	●	●
2	ADC	Spine	●	●	◐
3	ADC	Brain	●	●	◑
4	ADC	Brain	●	●	◯
5	ADC	Soft tissue	●	◐	●
6	ADC	Lung	●	◐	◑
7	ADC	Bone	●	◑	●
8	ADC	Pleura	●	◑	●
9	ADC	Kidney	●	◑	●
10	ADC	Pleura	●	◑	◐
11	ADC	Lung	●	◑	◐
12	ADC	Lung	●	◑	◐
13	ADC	Lung	●	◑	◯
14	ADC	Lung	●	◯	●
15	ADC	Lung	●	◯	●
16	ADC	Bronchus	●	◯	◯
17	ADC	Lung	◑	◑	●
18	ADC	Lung	◑	◑	●
19	ADC	Lung	◑	◑	●
20	ADC	Lung	◑	◑	◐
21	ADC	Lung	◑	◑	◯
22	ADC	Liver	◑	◑	◯
23	ADC	Pleura	◑	◯	●
24	ADC	Lung	◑	◯	●
25	ADC	Lung	◑	◯	◑
26	ADC	Bronchus	◯	◑	●
27	ADC	Lung	◯	◑	●
28	ADC	Brain	◯	◯	●
29	ADC	Brain	◯	◯	●
30	ADC	Lung	◯	◯	●
31	ADC	Lung	◯	◯	◐
32	SqCC	Kidney	●	●	◯
33	SqCC	Lung	●	◐	◯
34	SqCC	Brain	●	◑	●
35	SqCC	Lung	●	◑	◐
36	SqCC	Lung	●	◑	◐
37	SqCC	Bronchus	●	◑	◯
38	SqCC	Lung	●	◯	◯
39	SqCC	Bronchus	◐	◯	◐
40	SqCC	Brain	◑	●	◑
41	SqCC	Lung	◑	●	◑

ADC, adenocarcinoma; SqCC, squamous cell carcinoma. ^◯^negative in primary tumor and metastases; ^●^positive in primary tumor and metastases; ^◑^negative in primary tumor and positive in metastases; ^◐^positive in primary tumor and negative in metastases.

**Table IV:** Comparison of β-catenin, E-cadherin and Wnt1 expression between primary and metastatic tumors.

		β-catenin alteration			E-cadherin loss			Wnt1 overexpression		
				
Tumor type	Characteristics	+	−	P-value	+	−	P-value	+	−	P-value
Total (n=41)	Primary tumor	24	17	0.004	10	31	0.001	27	14	NS
	Metastatic tumor	34	7		26	15		23	18	
ADC (n=31)	Primary tumor	16	15	0.003	6	25	0.002	23	8	NS
	Metastatic tumor	25	6		19	12		20	11	
SqCC (n=10)	Primary tumor	8	2	NS	4	6	NS	4	6	NS
	Metastatic tumor	9	1		7	3		3	7	

ADC, adenocarcinoma; SqCC, squamous cell carcinoma; NS, not significant.

**Table V:** Comparison of β-catenin vs. E-cadherin and Wnt1 expression in adenocarcinomas.

		E-cadherin expression					Wnt1 expression				
				
Characteristic		◯	◑	◐	●	P-value	◯	◑	◐	●	P-value
β-catenin expression	◯	4	2	0	0	<0.001	0	0	1	5	NS
	◑	3	6	0	0		2	1	1	5	
	◐	0	0	0	0		0	0	0	0	
	●	3	7	2	4		3	2	4	7	

NS, not significant. ^◯^negative in primary tumor and metastases; ^●^positive in primary tumor and metastases; ^◑^negative in primary tumor and positive in metastases; ◐ Positive in primary tumor and negative in metastasis.
